# Prognostic impacts of glucocorticoid treatment in patients with polymyalgia rheumatica and giant cell arteritis

**DOI:** 10.1038/s41598-021-85857-4

**Published:** 2021-03-18

**Authors:** Amir Emamifar, Torkell Ellingsen, Anne Pernille Hermann, Søren Hess, Oke Gerke, Ziba Ahangarani Farahani, Per Syrak Hansen, Inger Marie Jensen Hansen, Peter Thye-Rønn

**Affiliations:** 1 Department of Clinical Research, Faculty of Health Sciences, University of Southern Denmark, Odense, Denmark; 2 Diagnostic Center, Svendborg Hospital, OUH, Baagøes Allé 15, 5700 Svendborg, Denmark; 3 Department of Rheumatology, Svendborg Hospital, OUH, Svendborg, Denmark; 4 OPEN, Odense Patient Data Explorative Network, Odense University Hospital, Odense, Denmark; 5 Rheumatology Research Unit, Odense University Hospital and University of Southern Denmark, Odense, Denmark; 6 Department of Endocrinology, Odense University Hospital, Odense, Denmark; 7 Department of Radiology and Nuclear Medicine, Hospital of Southwest Jutland, Esbjerg, Denmark; 8 Faculty of Health Sciences, Institute of Regional Health Research, University of Southern Denmark, Odense, Denmark; 9 Research Unit of Clinical Physiology and Nuclear Medicine, Department of Clinical Research, University of Southern Denmark, Odense, Denmark; 10 Department of Nuclear Medicine, Odense University Hospital, Odense, Denmark

**Keywords:** Vasculitis syndromes, Arterial stiffening, Osteopetrosis

## Abstract

Identifying comorbidities in polymyalgia rheumatica/giant cell arteritis (PMR/GCA) is crucial for patients’ outcomes. The present study aimed to evaluate the impact of the inflammatory process and glucocorticoid treatment on aortic arterial stiffness and body composition in PMR/GCA. 77 patients with newly diagnosed PMR/GCA were treated with oral glucocorticoids and followed for 40 weeks. Aortic pulse wave velocity (PWV) was measured at baseline and during the follow-up period and compared to the results of temporal artery biopsy (TAB) and 18F-FDG PET/CT. Body composition was assessed by total body DXA at baseline and the end of the study. Of 77 patients (49 (63.6%) female, mean of age: (71.8 ± 8.0)), 64 (83.1%) had pure PMR, 10 (13.0%) concomitant PMR and GCA, and 3 (3.9%) pure GCA. Compared to baseline values, aortic PWV was initially decreased at week 16 (p = 0.010) and remained lower than baseline at week 28 (p = 0.002) and week 40 (p < 0.001), with no association with results of TAB and 18F-FDG PET/CT. Aortic PWV was significantly associated with age, male gender, left systolic and diastolic blood pressure, right diastolic blood pressure, and CRP. Total bone mineral content (BMC) was decreased in both genders (p < 0.001), while fat mass (FM) was significantly increased (p < 0.001). However, lean body mass did not significantly change during the study. Changes in FM were correlated with cumulative prednisolone dose (rho: 0.26, p = 0.031). Glucocorticoid treatment of patients with PMR/GCA had several prognostic impacts. Arterial stiffness was decreased due either to the treatment or a reduction in the inflammatory load. Additionally, treatment led to changes in body composition, including a decrease in BMC and FM excess.

## Introduction

The recent European League Against Rheumatism (EULAR) recommendations for the management of polymyalgia rheumatica (PMR) and giant cell arteritis (GCA) underline the importance of screening for disease- and treatment-related comorbidities such as osteoporosis (and particularly recent fractures), cardiovascular disease, diabetes and dyslipidaemia^1,2^. These comorbidities are commonly observed in PMR/GCA and present additional challenges for clinicians. Identifying comorbidities at the time of diagnosis, before initiation of the glucocorticoid treatment, and underlying mechanisms and long-term complications is crucial and has been the subject of growing research efforts.

Arterial stiffness is an early and recognized risk factor for atherosclerosis^3,4^. It is a significant predictor for future cardiovascular disease as well as all-cause mortality, even in those without overt cardiovascular disease^4,5^. Arterial stiffness is caused by a generalized process of vascular aging that involves small to large arteries, and is increased in the presence of traditional cardiovascular risk factors^4,6^. Accumulating evidence suggests that the inflammatory process shares several pathogenic mechanisms with atherosclerosis and has a role in the initiation and progression of atherosclerosis^7–9^. This process in PMR/GCA can be explained by a complex interaction between inflammation, glucocorticoid treatment, and other individual risk factors, e.g. dyslipidemia, hypertension, obesity, and smoking^10^. Arterial stiffness is most frequently defined by aortic pulse wave velocity (PWV) and augmentation index (AIx) measured non-invasively using applanation tonometry, which is considered the gold standard technique to assess arterial stiffness^11^.

Another consequence of the inflammatory process together with glucocorticoid treatment is a decline in bone mass as well as a change in body composition^12–14^. Body composition refers to a three-compartment human body model consisting of bone mineral content (BMC), fat mass (FM), and lean body mass (LBM)^15^. While loss of bone mass puts patients at risk of future fractures, a decrease in LBM may contribute to the general debilitation of patients due to an increased fall tendency^16^. Glucocorticoid treatment alters energy metabolism by increasing protein breakdown and decreasing synthesis, promoting fat accumulation and fat redistribution from peripheral to central tissues, resulting in loss of LBM and increase in FM^13,17^. Increased FM is associated with cardiovascular disease and several metabolic morbidities, e.g. insulin resistance and type 2 diabetes mellitus^18^. Changes in body composition have previously been described in several rheumatic diseases, e.g. rheumatoid arthritis (RA) and systemic lupus erythematosus (SLE)^17,19–22^. However, data on this topic in PMR/GCA are scarce.

In light of the above considerations, the present study aimed to evaluate the impact of glucocorticoid treatment and the inflammatory process in PMR/GCA on aortic arterial stiffness and body composition at diagnosis and during a 40-week follow-up period.

## Materials and methods

### Study design and setting

This is a longitudinal cohort study. The study was performed at the Diagnostic Center in collaboration with the Section of Rheumatology at Svendborg Hospital, Svendborg, Denmark, between February 2018 and December 2019. Total body dual energy X-ray (DXA) scans were undertaken at the osteoporosis clinic, Odense University Hospital. Ethical approval was granted by the Regional Ethics Committee of the Region of Southern Denmark (identification number: S-20160098) and the Danish Data Protection Agency (J.nr 16/40522). All examinations were performed in accordance with relevant local guidelines and the informed consent was obtained from all included patients. The study was also registered at ClinicalTrials.gov (Identifier: NCT02985424, first posted on 07/12/2016).

### Participants

80 consecutive patients with newly suspected PMR, GCA, or concomitant PMR and GCA were included in the study. Inclusion and exclusion criteria have previously been described^23,24^. Briefly, PMR patients met the following criteria: 1. Age ≥ 50 years; 2. Bilateral shoulder or hip pain; 3. Morning stiffness > 45 min; 4. Elevated erythrocyte sedimentation rate (ESR) and/or C-reactive protein (CRP) and 5. Disease duration > 2 weeks. In case of Cranial-GCA (C-GCA), 1. Age ≥ 50 years; 2. Elevated ESR and/or CRP; 3. Scalp tenderness; 4. Vision disturbances; 5. Headache (new or changed); 6. Jaw claudication and 7. Tenderness of the temporal arteria were considered. Furthermore, patients with clinical suspicion of Large Vessel-GCA (i.e., upper extremity claudication and upper extremity blood pressure discrepancies) were also eligible for inclusion. Patients were excluded from the study if they met one of the below mentioned criteria:

1. Infections, malignancy, or any other conditions that prednisolone was unsuitable to initiate.
2. Contraindication to 18F-fluorodeoxyglucose positron emission tomography/computed tomography (18F-FDG PET/CT) i.e. blood glucose > 145 mg/dL after 6 h fasting.
3. Initiation of steroid treatment more than 3 days prior to 18F-FDG PET/CT.
4. Inability to provide informed consent.
5. Patients with dementia or inability to communicate in Danish.

All patients were treated with oral glucocorticoids according to current national guidelines with 20–30 mg/day in the case of PMR and up to 75 mg/day when GCA was suspected^23^. In addition, all patients received calcium/vitamin D supplementation alone or together with bisphosphonates if there were signs of osteopenia or osteoporosis on DXA according to current national guidelines. Patients were seen at baseline (visit 1), week 4 (visit 2), week 16 (visit 3), week 28 (visit 4), and week 40 (visit 5). The follow-up period was 40 weeks because previous studies have reported that this time period is sufficient to ensure a certain diagnosis^25,26^.

### Data collection

Patients’ demographics, clinical data, Charlson comorbidity index score^27^, and laboratory tests at baseline and follow-up visits were collected and managed by means of REDcap (Research Electronic Data Capture), which is a secure, web-based software platform designed to support data capture for research studies at the Open Patient data Explorative Network^28^. Cardiovascular disease risk prediction was assessed by Framingham risk score at baseline, which classifies the patients into three risk categories: low (< 10% risk of an event in 10 years), intermediate (10% to 20%), and high (> 20%) according to age, gender, smoking status, systolic blood pressure, total cholesterol, high-density lipoprotein cholesterol, antihypertensive treatment and presence of diabetes or known vascular disease^29^.

A unilateral temporal artery biopsy (TAB) was also performed in all included patients at baseline. TAB was considered positive if signs of active arteritis or healed arteritis were detected on pathologic examination.

Every included patient underwent an 18F-FDG PET/CT either before or in the case of GCA within 3 days of initiation of glucocorticoid treatment^23,24^. Based on previously described methodology, FDG uptakes in 8 paired articular/periarticular sites and 14 arterial segments were described visually based on a 4-point visual grading scale (VGS) with 0 = no uptake; 1 = slight but not negligible uptake, lower than liver uptake; 2 = intermediate uptake, equivalent to liver uptake; 3 = high-grade uptake, higher than liver uptake. Two pathologic cutoff values of VGS ≥ 3 and VGS ≥ 2 were used to analyze the results of 18F-FDG PET/CT^24^. Total PMR and GCA scores were defined as the sum of VGS at each articular/periarticular site or arterial segment.

### Aortic PWV analysis

Aortic PWV (m/s) and AIx (%) were measured by applanation tonometry using the Sphygmocor device (AtCor Medical, Sydney, Australia) by the same observer, who was blinded to 18FDG-PET/CT results, in every patient to avoid any inter-observer variability. All PWV measurements were undertaken after at least 10 min’ rest. Aortic PWV was calculated by measuring the surface distance between the two recording sites, carotid and femoral, and recording the sequential waveforms at the right common carotid and femoral arteries. Pulse transit time was calculated with the aid of a simultaneously recorded electrocardiogram as a common reference. Aortic PWV was then calculated automatically from measurements of pulse transit time and the distance between the two sites, with higher levels suggesting greater arterial stiffness. AIx was defined as the difference between the first and second peaks of the central arterial waveform expressed as a percentage of the pulse pressure and standardized to a heart rate of 75 beats per minute (AIx75 (%)), since AIx is influenced by alteration in heart rate.

### Total body DXA

Body composition was measured by total body DXA (Hologic Discovery QDR, Scanner ID: 82245) at baseline (visit 1), before or within 2 weeks after treatment initiation, and subsequently at week 40 (visit 5). FM, LBM, and BMC were assessed. FM was expressed as absolute numbers in grams and as a percentage of total body weight. Fat-free mass (FFM) was the sum of LBM and BMC. The fat mass index (FMI) and fat-free mass index (FFMI) were calculated as follows: FMI = FM/height^2^ and FFMI = FFM/height^2^. Data from a Swiss population of 5635 healthy adults (2986 men and 2649 women, age from 24 to 98 years) were used to categorize patients into the following groups: obese: FMI > 90th percentile, lean: FFMI < 10th percentile, wasted: FFMI < 10th percentile and FMI < 25th percentile, and cachectic: FFMI < 10th percentile and FMI > 25th percentile^30^. Osteopenia and osteoporosis were defined as a T score less than − 1.0 and greater than − 2.5 (− 1.0 < T score <  − 2.5) and osteoporosis as a T score ≤  − 2.5^31^.

### Partial patient and public involvement

The present study was supported by a patient advisory group that provided input to the research questions. Patients partnered with us regarding the design of the study, the informational material to support the intervention, and the burden of the intervention from the patient’s perspective, for instance, number of blood samples and time between blood samples. However additional patient involvement was difficult due to the technical nature of the methods used in the study. All participants will be informed of the trial results by mail. The study results will be disseminated to the public through online media.

### Statistical analysis

Data are presented as frequencies (percentages), mean ± standard deviation (SD), or median (interquartile range (IQR)) depending on data type and distribution. Correlation analysis was performed by Pearson’s correlation (r) or Spearman’s rank correlation (rho). A comparison of two paired binary variables was performed using McNemar’s test. The comparison of continuous variables was performed by Student’s t-test or Wilcoxon rank-sum test (Mann–Whitney U test), if unpaired, and Wilcoxon signed-rank test or paired Student’s t-test, if paired, depending on the assumption of normally distributed data. The Kruskal–Wallis test or analysis of variance was used when more than two groups were compared. Analysis of PWV repeated measurements over time was done by means of mixed linear model considering aortic PWV as the outcome variable and age, gender, clinical diagnosis, blood pressure, and CRP as fixed factors. Data from a European cross-sectional study in 11,092 individuals (Mean age (year) ± SD: 50 ± 17, Gender Male/Female: 5520/5572) without overt cardiovascular disease and diabetes mellitus were used as reference values for PWV in a subgroup of our patients without these comorbidities^32^. A p value was considered as significant if p < 0.05. No method of imputation was used for missing data. Statistical analysis was performed using STATA version 16.0 (StataCorp, College Station, TX, USA).

## Results

Of 80 included patients, 3 patients were diagnosed with seronegative RA during the follow-up period. Statistical analyses were performed in 77 patients. Among 77 patients, 64 (83.1%) patients were diagnosed with pure PMR, 10 (13.0%) patients with concomitant PMR and GCA, and 3 (3.9%) with pure GCA, the clinical diagnoses being confirmed during the follow-up period. The mean age of the patients was 71.8 ± 8.0 years and 49 (63.6%) were women. Baseline demographic data have previously been published in detail^24,33^. Clinical data together with laboratory results at visit 1 to visit 5 are summarized in Table 1. The total number of relapse during the study and cumulative prednisolone dose were (mean ± SD: 0.6 ± 0.9, median (IQR): 0 (0 to 1), min: 0, max: 5) and (mean ± SD: 2995.1 ± 1269.7, median (IQR): 2438.7 (2193.7 to 3420), min: 1226.25, max: 7511.25), respectively. 69 (89.6%) patients out of 77 completed the study. The numbers of patients who withdrew from the study together with the reason for withdrawal are summarized in Supplementary Table S1 (disposition table). Of 70 patients who agreed to performance of TAB, TAB was positive in 7 (10%) patients: active arteritis n = 4 (5.7%) and healed arteritis n = 3 (4.3%).

**Table 1:** Clinical data at baseline, week 4, week 16, week 28, and week 40.

Variables	Baseline*	Week 4	Week 16	Week 28	Week 40
Left systolic blood pressure, mm/Hg	137 (127–152)	139.6 ± 20.7 0.76^1^**	137 (128–151) 0.85^1^	136.4 ± 17.5 0.18^1^	130.3 ± 18.6 ** < 0.001**^**1**^
Left diastolic blood pressure, mm/Hg	82.3 ± 10.5	82.7 ± 10.5 0.67^2^	83.1 ± 11.7 0.60^2^	81.5 ± 10.5 0.61^2^	79.1 ± 11.5 **0.009**^**2**^
Right systolic blood pressure, mm/Hg	137 (124.5–147)	137.7 ± 21.9 0.76^1^	135 (123–150) 0.99^1^	133.0 ± 18.0 0.22^1^	130.7 ± 19.6 **0.005**^**1**^
Right diastolic blood pressure, mm/Hg	80.3 ± 11.2	81.1 ± 11.4 0.46^2^	81.8 ± 11.9 0.30^2^	80.1 ± 10.6 0.91^2^	78.4 ± 10.3 0.24^2^
Left radial pulse, per minute	76.4 ± 13.7	69.7 ± 10.3 ** < 0.001**^**2**^	71.5 (63–79) **0.001**^**1**^	70.7 ± 11.7 ** < 0.001**^**2**^	71.4 ± 12.1 **0.001**^**2**^
Right radial pulse, per minute	75.6 ± 13.9	68.8 ± 10.7 ** < 0.001**^**2**^	72.8 ± 12.4 **0.031**^**2**^	70.0 ± 11.6 ** < 0.001**^**2**^	71.9 ± 11.8 **0.005**^**2**^
Charlson comorbidity index score	3 (2–4)	3 (2–4) 0.64^1^	3 (2–4) 0.42^1^	3 (2–4) 0.71^1^	3 (2.5–4) **0.032**^**1**^
Patients pain VAS score	75 (50–82.5)	0 (0–15) ** < 0.001**^**1**^	10 (0–17) ** < 0.001**^**1**^	10 (0–25) ** < 0.001**^**1**^	15 (5–40) ** < 0.001**^**1**^
Patients global VAS score	80 (60–90)	1 (0–20) ** < 0.001**^**1**^	5 (0–20) ** < 0.001**^**1**^	10 (0–40) ** < 0.001**^**1**^	17.5 (0–40) ** < 0.001**^**1**^
Physician global VAS score	30 (25–40)	5 (2–5) ** < 0.001**^**1**^	5 (2–5) ** < 0.001**^**1**^	3 (2–5) ** < 0.001**^**1**^	5 (3–10) ** < 0.001**^**1**^
Hemoglobin, mmol/L [8.3–10.5]	7.6 (7.2–8.1)	8.2 (7.8–8.7) ** < 0.001**^**1**^	8.5 (8.1- 9) ** < 0.001**^**1**^	8.4 ± 0.8 ** < 0.001**^**1**^	8.3 ± 0.8 ** < 0.001**^**1**^
Leucocytes, 10E9/L [3.50–8.80]	9.4 (8.05–11.2)	10.9 (9.4–13) ** < 0.001**^**1**^	10.1 ± 2.9 0.15^1^	9.5 ± 2.6 0.91^1^	8.8 ± 2.6 **0.008**^**1**^
ESR, mm [2–20]	53.5 (38–77)	8 (5–12) ** < 0.001**^**1**^	10 (6–14) ** < 0.001**^**1**^	9 (6–18) ** < 0.001**^**1**^	11 (6–23) ** < 0.001**^**1**^
CRP, mg/L [< 6.0]	37 (17–64)	1.9 (1.1–4.3) ** < 0.001**^**1**^	2.4 (1–5.8) ** < 0.001**^**1**^	2.3 (1–4.9) ** < 0.001**^**1**^	3.3 (1.4–8.1) ** < 0.001**^**1**^
Fibrinogen, µmol/L [5.2–12.6]	14.9 (13–17.5)	8.6 ± 1.5 ** < 0.001**^**1**^	9.4 (8.2–10.6) ** < 0.001**^**1**^	9.4 (8.4–10.8) ** < 0.001**^**1**^	10.5 ± 2.1 ** < 0.001**^**1**^
Numbers of relapse since previous visit, sum (median (interquartile range))***	–	0 (0 (0–0))	12 (0 (0–0)) ** < 0.001**^**1**^	14 (0 (0–0)) **0.002**^**1**^	18 (0 (0–1)) ** < 0.001**^**1**^
Prednisolone initial (baseline) or actual (follow-up) dose	20 (20–30)	17.5 (17.5–25) ** < 0.001**^**1**^	7.5 (7.5–10) ** < 0.001**^**1**^	5 (3.7–6.2) ** < 0.001**^**1**^	2.5 (0–3.7) ** < 0.001**^**1**^
Cumulative prednisolone dose since previous visit***	–	632.5 (475–1075)	1071.2 (945–1471.2) ** < 0.001**^**1**^	525 (450–657.5) ** < 0.001**^**1**^	265 (163.7–428.7) ** < 0.001**^**1**^

*VAS* visual analogue scale, *ESR* erythrocyte sedimentation rate, *CRP* C-reactive protein.

p values marked in bold represent significant results.

*Baseline data are gathered prior to initiation of glucocorticoids.

**p values are calculated by comparison of baseline data with week 4, 16, 28 and 40.

***p values are calculated by comparison of week 4 with week 16, 28 and 40.

^1^Wilcoxon signed-rank test.

^2^Paired Student’s t-test.

### Framingham risk score

Supplementary Table S2 summarized the cardiovascular risk factors for the patients. The risk stratification showed that 19.7%, 36.8% and 43.4% of the included patients were classified as of low, intermediate and high -risk at baseline, respectively.

### 18F-FDG PET/CT results

The means ± SDs of the total PMR and GCA scores were 12.5 ± 5.9 and 0.8 ± 2.0, respectively. With a pathologic cutoff value of ≥ 3, 55 (71.4%) patients had signs of PMR activity, 2 (2.6%) GCA activity, 1 (1.3%) PMR and GCA activity, and 19 (24.7%) had neither PMR nor GCA activity. On the other hand, with a pathologic cutoff value of ≥ 2, 58 (75.3%) patients showed PMR activity, 3 (3.9%) GCA activity, 9 (11.7%) PMR and GCA activity, and 7 (9.1%) had neither PMR nor GCA activity^24^.

### Aortic PWV analysis

Male patients had a higher aortic PWV than female patients at baseline (12.9 (11.2 to 14.45) vs 11.5 (10.5 to 12.9) p = 0.023). Results of aortic PWV analysis at visit 1 to visit 5 are summarized in Table 2. Aortic PWV did not show any significant correlation with baseline AIx (rho = 0.15, p = 0.21) and AIx75 (rho: 0.12, p = 0.30) (Supplementary Table S3).

**Table 2:** PWV analysis at baseline, week 4, week 16, week 28, and week 40.

Variables	Baseline	Week 4	Week 16	Week 28	Week 40
Aortic SP, mmHg	128.5 ± 12.8	129.4 ± 15.4 0.70*^1^	128 (118–140) 0.61^2^	126.4 ± 12.6 0.31^1^	123.6 ± 12.7 **0.010**^**1**^
Aortic DP, mmHg	83.4 ± 10.6	81.9 ± 9.8 0.19^1^	84.7 ± 11.2 0.22^1^	83 (76–90) 0.24^2^	81.1 ± 10.3 **0.021**^**1**^
Aortic PP, mmHg	45.0 ± 12.1	47.5 ± 12.8 0.07^1^	45.2 ± 11.0 0.79^1^	43 (36.5–50) 0.82^2^	42.5 ± 10.8 0.20^1^
MAP, mmHg	101.3 ± 10.4	99.9 ± 11.0 0.28^1^	101.9 ± 12.2 0.51^1^	99.3 ± 10.9 0.16^1^	97.4 ± 10.5 **0.004**^**1**^
Aortic AP, mmHg	10 (6–15)	11.6 ± 7.4 0.14^2^	10 (7–14) 0.88^2^	9.9 ± 6.7 0.99^2^	9.7 ± 6.9 0.99^2^
AIx, %	21.6 ± 12.2	23.1 ± 12.1 0.21^1^	22.8 ± 11.2 0.28^1^	21.6 ± 12.8 0.71^1^	21.4 ± 13.0 0.75^1^
AIx75, %	21.4 ± 12.4	20.3 ± 11.5 0.64^1^	20.5 ± 11.7 0.87^1^	19.5 ± 12.7 0.41^1^	19.7 ± 12.3 0.47^1^
HR, bpm	67.9 ± 12.6	62.7 ± 10.4 ** < 0.001**^**1**^	64.7 ± 10.4 **0.020**^**1**^	63.7 ± 9.5 **0.007**^**1**^	65.3 ± 10.0 **0.023**^**1**^
Pulse transit time, ms	49 (44–55)	51.0 ± 8.4 0.16^2^	51.2 ± 9.1 0.07^2^	52.0 ± 9.2 **0.027**^**2**^	51.6 ± 8.7 **0.022**^**2**^
PWV, m/s	11.8 (10.7–13.6)	11.9 ± 1.9 0.20^2^	11.5 (10.1–12.9) **0.010**^**2**^	11.5 ± 2.1 **0.002**^**2**^	11.4 ± 2.0 ** < 0.001**^**2**^

*SP* systolic pressure, *DP* diastolic pressure, *PP* pulse pressure, *MAP*: mean arterial pressure, *AP* augmented pressure, *AIx* aortic augmentation index, *AIx75* aortic augmentation index standardized to a heart rate of 75 beats per minute, *HR* heart rate, *PWV* pulse wave velocity.

p values marked in bold represent significant results.

*p values are calculated by comparison of baseline data with week 4, 16, 28 and 40.

^1^Paired Student’s t-test.

^2^Wilcoxon signed-rank test.

**Figure 1:**
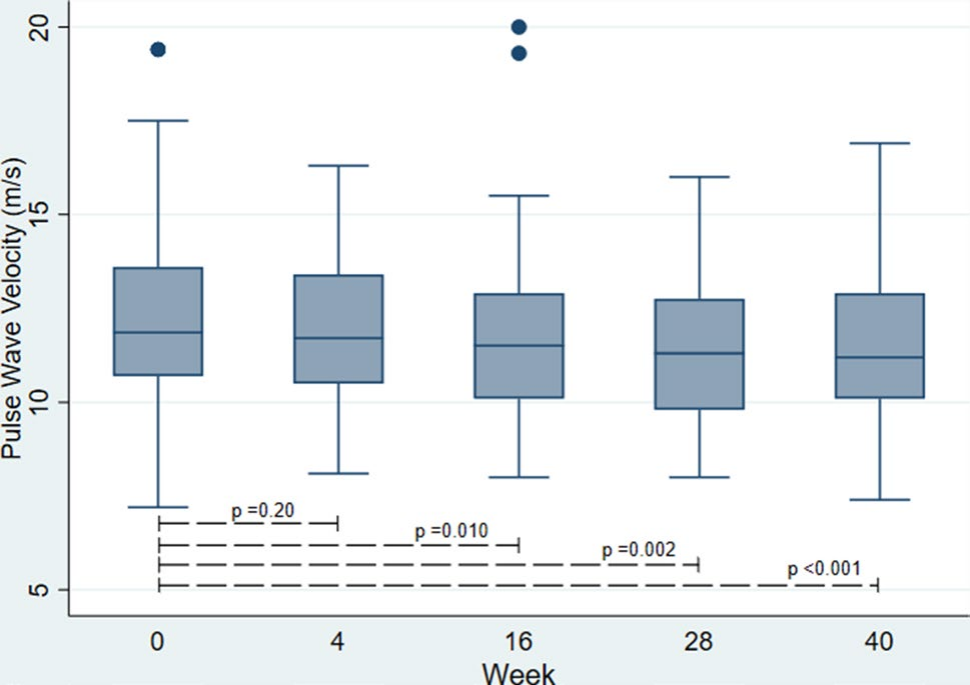
Box plot of aortic PWV at baseline (visit 1), week 4 (visit 2), week 16 (visit 3), week 28 (visit 4), and week 40 (visit 5) including p values from pairwise comparisons. (The figure is graphed using Stata version 16.0 (StataCorp LLC, College Station, TX, USA, https://www.stata.com/)).

Compared to the baseline (visit 1), aortic PWV was initially decreased at week 16 (visit 3, p = 0.010) and remained below baseline at week 28 (visit 4, p = 0.002) and week 40 (visit 5, p < 0.001) (Fig. 1). No significant difference was found between baseline aortic PWV (visit 1) and aortic PWV at week 4 (visit 2, p = 0.20). AIx and AIx75 did not significantly differ from baseline values during the follow-up period (Supplementary Fig. S2A,B).

There were no significant differences in aortic PWV between patients with low, intermediate and high future cardiovascular risk at baseline (p = 0.09), week 4 (p = 0.11) and week 28 (p = 0.053), although it was tended to be higher in the intermediate- and high-risk patients (Supplementary Fig. S3). At week 16 and week 40, between groups analyses showed that aortic PWV was significantly higher in patients with high (high- vs low-risk p = 0.026 (week 16) and 0.003 (week 40)) and intermediate future cardiovascular risk (intermediate- vs low-risk p = 0.010 (week 16) and 0.009 (week 40)) compared to low-risk patients.

With regard to TAB and 18F-FDG PET/CT results, there were no significant differences in AIx, AIx75, and aortic PWV between the groups (Supplementary Table S4). Furthermore, baseline aortic PWV, Aix, and AIx75 did not show any significant correlation with total PMR and GCA scores (Supplementary Table S5).

In the most robust mixed model analysis, aortic PWV was significantly associated with age (coefficient: 0.096, p < 0.001), male gender (coefficient: 1.040, p = 0.009), left systolic blood pressure (coefficient: 0.016, p = 0.036), left diastolic blood pressure (coefficient: 0.030, p = 0.016), right diastolic blood pressure (coefficient: 0.023, p- = 0.041), and CRP (coefficient: 0.005, p- = 0.029) (Table 3). Framingham risk score was not significantly associated with aortic PWV in the regression model. Besides, when the results of TAB and 18F-FDG PET/CT (cutoff values ≥ 3 and ≥ 2) were sequentially added to the model, they did not achieve statistical significance.

**Table 3:** Mixed model analysis considering PWV as the outcome variable.

Variables	Coefficient	Standard error	95% Confidence interval	p value
Age	0.096	0.026	0.045–0.147	** < 0.001**
Male gender	1.040	0.397	0.261–1.818	**0.009**
**Clinical diagnosis**				
Pure PMR	− 0.710	0.591	− 1.869–0.448	0.23
Pure GCA	− 0.888	1.143	− 3.129–1.353	0.44
Left systolic BP	0.016	0.008	0.001–0.031	**0.036**
Left diastolic BP	0.030	0.012	0.005–0.054	**0.016**
Right systolic BP	0.003	0.006	− 0.008–0.014	0.59
Right diastolic BP	0.023	0.011	0.001–0.046	**0.041**
CRP	0.005	0.002	0.000–0.009	**0.029**

*BP* blood pressure, *CRP* C-reactive protein.

p values marked in bold represent significant results.

Comparison of the median of baseline PWV values in a subgroup of our patients without overt cardiovascular disease and diabetes mellitus (n = 65) with values in age- and blood pressure-matched individuals from the European reference population^32^, suggested that the baseline aortic PWV was probably higher in PMR/GCA patients than in the reference population (Supplementary Table S6, Supplementary Fig. S4). However, a definite comparison could not be made because of too few observations in several groups and lack of a formal control group.

### Total body DXA measurements

Results of total body DXA at baseline (visit 1) and week 40 (visit 5) are summarized in Table 4. Total BMC was significantly decreased in both male (p ≤ 0.001) and female (p ≤ 0.001) patients. Additionally, FM was significantly increased in both male (p ≤ 0.001) and female (p ≤ 0.001) patients. However, LBM did not significantly change during the study in male (p = 0.17) and female (p = 0.67) patients. Changes in FM were significantly correlated with cumulative prednisolone dose (rho: 0.26, p = 0.031) and was greater in male patients (2822.9 (2263 to 4956) vs 2138.5 (254.6 to 3486.9) p = 0.049). There were no significant correlations between cumulative prednisolone dose and changes in total BMC (rho: − 0.17, p = 0.16) and LBM (rho: − 0.15, p = 0.22).

**Table 4:** Body composition assessed by DXA at baseline and week 40.

Variables	Male			Female		
	Baseline	Week 40	p value	Baseline	Week 40	p value
**Total body**						
BMI, kg/m^2^	27 (24 to 28.1)	28.4 (26.1–29.1)	** < 0.001**^**1**^	24.4 (21.5 to 28.2)	25.0 (21.8 to 28.4)	** < 0.001**^**1**^
Area, cm^2^	2291.4 ± 182.3	2282.7 ± 216.8	0.14^2^	1879.4 ± 190.3	1857.4 ± 188.5	**0.020**^**2**^
BMC, g	2481.4 (2219.2 to 2951.9)	2390.9 (2080.0 to 2792.7)	** < 0.001**^**1**^	1822.0 (1684.4 to 2064.4)	1757.9 (1617.0 to 1943.3)	** < 0.001**^**1**^
BMD, g/cm^2^	1.1 ± 0.1	1.0 (1.0 to 1.2)	** < 0.001**^**1**^	1.0 ± 0.1	1.0 (0.9 to 1.0)	** < 0.001**^**1**^
FM, g	26,145.8 (20,786–28,785.4)	28,461.6 (23,503.6–32,560.5)	** < 0.001**^**1**^	23,704 (20,620.5–31,666.3)	25,524.6 (21,310.1 to 32,929.7)	** < 0.001**^**1**^
LBM, g	54,003.1 ± 5952.2	54,889.8 (51,133.8–56,711.7)	0.17^1^	38,700.7 ± 5798.7	37,804.9 (34,045.6 to 41,308.5)	0.67^1^
FFM, g	56,570.6 ± 6168.1	57,064.7 (53,087.1 to 59,029)	0.11^1^	40,587.6 ± 6009.9	39,538.7 (35,909.8 to 43,538.6)	0.47^1^
Total, g	81,821 ± 10,956.0	84,940.0 ± 12,120.1	** < 0.001**^**2**^	67,067.9 ± 14,610.8	68,436.0 ± 16,634.8	**0.003**^**2**^
FM, %	30.5 ± 4.6	33.0 ± 5.1	** < 0.001**^**2**^	38.5 ± 6.7	40.3 ± 7.5	** < 0.001**^**2**^
FMI, kg/m^2^	8.2 (6.7 to 9.4)	9.7 (8 to 10.2)	** < 0.001**^**1**^	9.2 (8.3 to 11.8)	10 (8.3 to 12.4)	** < 0.001**^**1**^
FFMI, kg/m^2^	18.4 ± 1.6	18.3 ± 1.4	0.18^2^	15.4 ± 1.6	15.3 ± 1.7	0.62^2^
T score	− 0.9 ± 1.4	− 1.3 ± 1.4	** < 0.001**^**2**^	− 1.5 ± 1.3	− 1.9 ± 1.4	** < 0.001**^**2**^
Z score	− 0.3 ± 1.2	− 0.6 ± 1.2	** < 0.001**^**2**^	− 0.1 ± 1.1	− 0.3 ± 1.0	** < 0.001**^**2**^
**Hip**						
Area, cm^2^	46.4 ± 4.4	46.5 ± 4.3	0.52^2^	36.3 ± 4.0	35.8 ± 3.9	0.09^2^
BMC, g	41.7 (36.0 to 48.1)	40.5 (37.0 to 47.9)	0.37^1^	26.7 (23.8 to 30.2)	26.6 (23.4 to 30.4)	0.29^1^
BMD, g/^cm2^	0.9 (0.8 to 1.0)	0.9 (0.8 to 1.0)	0.26^1^	0.7 (0.7 to 0.8)	0.8 (0.7 to 0.8)	**0.010**^**1**^
T score	− 0.9 (− 1.7 to − 0.3)	− 0.9 (− 1.5 to − 0.3)	0.44^1^	− 1.6 (− 2 to − 1.1)	− 1.5 (− 2 to − 0.8)	**0.013**^**1**^
Z score	− 0.2 (− 0.8 to 0.3)	− 0.1 (− 0.6 to 0.3)	0.25^1^	− 0.1 (− 0.4 to 0.5)	0.1 (− 0.3 to 0.8)	** < 0.001**^**1**^
**Spine**						
Area, cm^2^	62.7 ± 15.8	62.1 ± 16.1	0.77^2^	52.6 ± 11.9	51.8 ± 12.1	0.11^2^
BMC, g	59.6 (49.4 to 74.4)	61.3 ± 21.1	0.12^1^	44.7 (34.9 to 57.0)	45.7 ± 15.1	0.44^1^
BMD, g/cm^2^	1.0 (0.8 to 1.0)	0.9 (0.8 to 1.0)	0.11^1^	0.9 (0.7 to 0.9)	0.9 (0.8 to 1.0)	**0.024**^**1**^
T score	− 1.1 (− 2 to − 0.3)	− 1.2 (− 2.1 to − 0.5)	0.11^1^	− 1.6 (− 2.6 to − 0.9)	− 1.7 (− 2.4 to − 0.8)	0.07^1^
Z score	0.0 ± 1.6	0.0 ± 1.6	0.26^2^	0.6 ± 1.3	0.7 ± 1.3	**0.009**^**2**^

*BMI* body mass index, *BMC* bone mineral content, *BMD* bone mineral density, *FM* fat mass, *LB*M lean body mass, *FFM* fat-free mass, *FMI* fat mass index, *FFMI* fat-free mass index.

^1^Wilcoxon signed-rank test.

^2^Paired Student’s t-test.

p values marked in bold represent significant results.

The prevalence of obese (p = 0.039) patients increased significantly during follow-up period (Table 5). Besides, the prevalences of osteopenia (p = 0.12), underlean (p = 0.62), wasted (p = 1.00) and cachexia (p = 1.00) were slightly increased, but it did not reach statistical significance.

**Table 5:** Prevalence of osteopenia and osteoporosis and underlean, obese, cachexia, and wasted during the study.

Variables	Baseline	Week 40	p value
Osteopenia, n (%)	30 (43.5%)	35 (50.7%)	0.12^1^
Osteoporosis, n (%)	19 (27.5%)	16 (23.2%)	0.25^1^
Osteopenia or osteoporosis, n (%)	49 (71.0%)	51 (73.9%)	0.62^1^
Obese, n (%)	20 (29.0%)	27 (39.1%)	**0.039**^**1**^
Underlean, n (%)	12 (17.4%)	14 (20.3%)	0.62^1^
Wasted, n (%)	1 (1.4%)	2 (2.9%)	1.00^1^
Cachexia, n (%)	11 (15.9%)	12 (17.4%)	1.00^1^

^1^McNemar’s test.

p values marked in bold represent significant results.

21 (28%) patients were treated with calcium/vitamin D supplementation alone, while 54 (72%) patients were treated with bisphosphonates in combination with calcium/vitamin D supplementation. Changes in total BMC (− 104.3 (− 211.0 to − 67.8) vs − 74.3 (− 122.7 to − 38.9), p = 0.08), hip BMC (− 0.3 (− 0.9 to 0.5) vs 0.3 (− 0.6 to 0.8), p = 0.14), and spine BMC (− 1.4 (− 4.4 to 1.0) vs 0.3 (− 0.8 to 1.32), p = 0.010) were negative and greater in those who were treated with calcium/vitamin D supplementation alone compared to those who received bisphosphonates in combination with calcium/vitamin D supplementation, although only changes in spine BMC reached statistical significance. Supplementary Fig. S5 illustrated changes in total BMC, as well as hip and spine T scores in those who were treated with calcium/vitamin D supplementation alone, compared to those who were treated with calcium/vitamin D supplementation together with bisphosphonates.

## Discussion

To the best of the authors’ knowledge, this is the first study of its kind investigating two important comorbidities, namely arterial stiffness and body composition, in patients with PMR/GCA in a 40-week longitudinal study. The decrease in aortic PWV was initially seen at week 16 (visit 3) and remained below the baseline value at week 28 (visit 4) and week 40 (visit 5). However, AIx and AIx75 did not change significantly during the study. In addition, the decrease in aortic PWV was independent of TAB and 18F-FDG PET/CT results. In the mixed model regression analysis, aortic PWV was significantly associated with age, male gender, left systolic blood pressure, left diastolic blood pressure, right diastolic blood pressure, and CRP. Comparison of the baseline PWV analysis in the present cohort with the age- and blood pressure-matched individuals from the European reference population suggested that the baseline aortic PWV in PMR/GCA patients tended to be higher than that in the reference population.

With regard to body composition, BMC decreased significantly in both genders during the study. Additionally, there was a significant increase in FM in both genders, which was significantly correlated with cumulative prednisolone dose. However, LBM did not change significantly during the study. Prevalence of obese patients was significantly higher at the end of the study (week 40) than at baseline. Those who were treated with calcium/vitamin D supplementation alone had a greater loss of total, hip, and spine BMC than those treated with supplementation and bisphosphonates, although only changes in spine BMC reached statistical significance.

PMR and GCA are inflammatory rheumatic diseases that frequently overlap each other. They share several features for instance genetic background and clinical manifestations and it is believed that PMR and GCA belong to the same disease entity, although it is difficult to draw a definite conclusion^34^. In this regard several authors suggest a disease spectrum where PMR is at one side of the spectrum, representing a milder subset, and GCA is at the other end of the spectrum, representing a severe subset of the disease^35^. Imaging findings also support this model by illustrating the synchronous findings of PMR and GCA in the patients^36^.

Previous research on arterial stiffness in PMR and/or large vessel vasculitis, GCA and Takayasu’s arteritis, is limited to a few studies^37–42^. In a study by Pieringer et al. in 13 patients with newly diagnosed PMR and 30 age- sex-matched subjects as a control group, AIx tended to be higher in the PMR group and improved significantly from 28.5 to 25.3 (p = 0.006) after 4 weeks of treatment with 25 mg/day prednisolone^37^. In another study by Schillaci et al. in 39 patients with PMR and 39 age-, sex-, and blood pressure-matched healthy subjects, aortic PWV was higher in patients with PMR than in control subjects (12.4 ± 4 vs 10.2 ± 2 m/s, p ≤ 0.01)^38^. Both aortic PWV (from 11.8 ± 3 to 10.5 ± 3 m/s, p = 0.015) and AIx75 (from 34 ± 7 to 29 ± 8, p = 0.012) were decreased after 4 weeks of treatment with prednisone at a dose of 15 mg/day, and the change in aortic PWV was significantly correlated with the percentage change in plasma CRP. Our results support these findings, although with a few differences. In our study, the decrease in aortic PWV was first seen after 16 weeks from diagnosis without any changes in AIx or AIx75 being found. This is probably due to the difference in the patient populations because patients in Schillaci et al.’s study were selected from PMR patients without risk factors that would interfere with large artery properties: diabetes mellitus, overt cardiovascular, renal disease, smokers, and patients receiving treatment with antihypertensive drugs or any vasoactive drugs. We collected real-world data in a patient population from a general rheumatology clinic that included patients with both PMR and GCA with comorbid conditions, which minimized possible selection bias. The effects of glucocorticoids on atherosclerosis and cardiovascular events are debated and not well understood. Although glucocorticoids have a well-recognized impact on insulin resistance, lipid profile, blood sugar, blood pressure, and obesity, all well-known risk factors for cardiovascular disease, an anti-atherosclerotic effect of glucocorticoid has been identified in animal models^43–45^. The underlying anti-atherosclerotic mechanism of glucocorticoids is thought to be related to the anti-inflammatory effect of glucocorticoids^46^. Whether the benefit of glucocorticoids on arterial stiffness by reducing the inflammatory state is a short-term effect or remains after cessation of treatment is a matter of interest and needs further research.

In our study, over 40% of the patients were categorized as a high-risk group for future cardiovascular events. In addition, aortic PWV was tended to be higher in the patients with high and intermediate future cardiovascular risk compared to the low risk patients. In this regard, in a retrospective, population-based incidence cohort comparing GCA with a cohort of age-, sex- and calendar year-matched patients without GCA, the overall Framingham risk score in the GCA cohort did not significantly differ from the non-GCA cohort^47^. In the present cohort, it was not possible for us to further elaborate on the results due to the lack of a control group. It is worth mentioning that, the evidence regarding the utility of Framingham score in PMR and GCA is scarce and additionally, it may underestimate the cardiovascular risk in the patients, as it is initially developed for predicting cardiovascular risk in the general population and its value is not well investigated in the patients with inflammatory diseases^48,49^.

Glucocorticoids are partly responsible for the loss of bone mass and changes in body composition seen in several rheumatic diseases like SLE or RA^17,19–22^. They have negative effects on BMC and are the principal cause for secondary osteoporosis^50^. The mechanism behind is mainly related to enhancing bone resorption by increasing the life span of osteoclasts together with decreasing bone formation^50^. Due to standard bone protection, calcium/vitamin D and/or bisphosphonates started together with glucocorticoid treatment, hip and spine T scores remained fairly stable during the study period. Furthermore, as we reported earlier, the adherence to osteoporosis medications in our PMR/GCA population was high^51,52^. Although previously identified, the effect of glucocorticoids, or the diseases themselves, on body composition in rheumatic diseases is often neglected^53^. Treatment with glucocorticoids can lead to an increase in FM and central obesity. These are unfavourable conditions that can induce activation of the inflammatory response by producing pro-inflammatory cytokines, such as TNF-a and IL-6^54^. Also, decreased physical function due to the disease itself, causes deterioration of the patient’s general condition and puts the patient at an increased risk of metabolic syndrome and cardiovascular events. Our results demonstrated a significant increase in FM in both genders, changes in FM being correlated with the cumulative prednisolone dose. These findings are in line with a previous study by Nordborg et al. in 24 patients with GCA (19 women and 5 men, 16 TAB positive) showed a significant increase in total body fat at 3, 6, 12, and 24 months compared to baseline values. Cumulative prednisolone dose was significantly correlated with FM in Nordborg et al.’s study^55^.

Our study has some limitations. Firstly, our patient population had several comorbid diseases, e.g. diabetes mellitus and hypertension. Although this minimized the selection bias, the comorbidities may have served as confounding factors and had varied impacts on our results. Secondly, the present study was observational by design and not a randomized controlled trial. Whether the changes in PWV and body composition are related to glucocorticoid use or the reduction in inflammatory load cannot be distinguished. However, due to the possible severe consequence of a placebo-controlled trial in PMR, this option was not applicable. Thirdly, cachexia in RA was first defined in 1992 and is different from the cachexia seen in cancer diseases^56^. Whether this term can be used interchangeably in PMR and GCA is unknown and needs to be investigated. The major strengths of our study were a relatively long follow-up period and the prospective design and the application and comparison of 18F-FDG PET/CT and aortic PWV analysis in terms of arterial stiffness. Furthermore, the study population represented a real-life PMR and GCA population with varied phenotypes of the disease, and thus our study has a high degree of generalizability.

In conclusion, our data demonstrated that treatment with glucocorticoids in patients with PMR/GCA had several prognostic impacts. Glucocorticoid treatment caused a decline in arterial stiffness, and this decline was independent of TAB and 18F-FDG PET/CT results. Additionally, it resulted in a decrease in BMC and an increase in FM. Anti-osteoporotic medications were beneficial and prevent further bone loss and should be initiated together with glucocorticoids. Lifestyle advice to reduce cardiovascular risk has also a preventive role in the management of the patients.

## Supplementary Information

Supplementary Figures.

Supplementary Tables.

## Data Availability

On request, an anonymised dataset will be shared. Provided permission is given from the Danish Data Protection Agency.
