# Supplementary material for: Prognostic impacts of glucocorticoid treatment in patients with polymyalgia rheumatica and giant cell arteritis
Source: Sci Rep. 2021 Mar 18;11:6220. doi: 10.1038/s41598-021-85857-4 (PMC7973518; doi:10.1038/s41598-021-85857-4)
Supplement: Supplementary file 1 — Supplementary Figures. [file 41598_2021_85857_MOESM1_ESM.docx]

**Prognostic impacts of Glucocorticoid treatment in patients with Polymyalgia Rheumatica and Giant Cell Arteritis.**

Amir Emamifar* (0000-0001-5329-1364), MD, PhD, [amir.emamifar@rsyd.dk](mailto:amir.emamifar@rsyd.dk), Torkell Ellingsen, (0000-0003-0426-4962), Clinical professor, PhD, [torkell.ellingsen@rsyd.dk](mailto:torkell.ellingsen@rsyd.dk), Anne Pernille Hermann, (0000-0001-6701-0981), Clinical associate professor, PhD, [pernille.hermann@rsyd.dk](mailto:pernille.hermann@rsyd.dk), Søren Hess (0000-0003-1249-133X), Clinical associate professor, [soren.hess@rsyd.dk](mailto:soren.hess@rsyd.dk), Oke Gerke (0000-0001-6335-3303), Professor, PhD, [Oke.Gerke@rsyd.dk](mailto:Oke.Gerke@rsyd.dk), Ziba Ahangarani Farahani, Senior consultant, [ziba.farahani2@rsyd.dk](mailto:ziba.farahani2@rsyd.dk), Per Syrak Hansen, Senior consultant, [Per.Syrak.Hansen@rsyd.dk](mailto:Per.Syrak.Hansen@rsyd.dk), Inger Marie Jensen Hansen (0000-0001-7283-9786), Former clinical associate professor, PhD, [imjh@carlhansen.dk](mailto:imjh@carlhansen.dk), Peter Thye-Rønn (0000-0002-7486-8086), Clinical associate professor, PhD, [Peter.Thye-Ronn@rsyd.dk](mailto:Peter.Thye-Ronn@rsyd.dk)


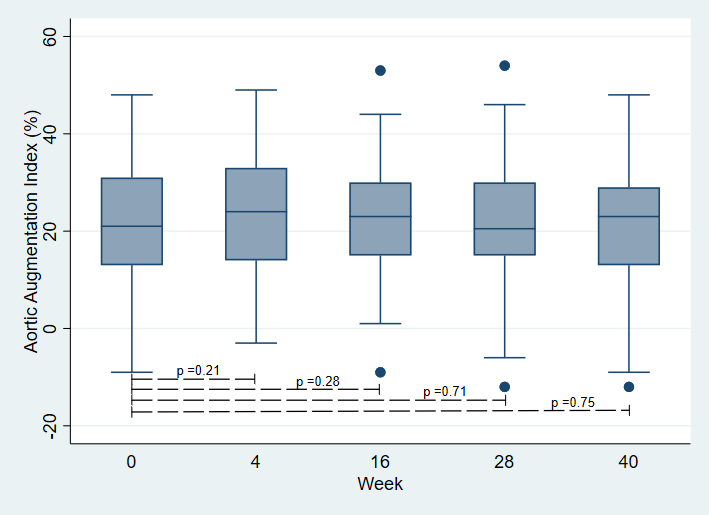


**Supplementary Figure 2 A:** Box plot of aortic augmentation index (AIx) at baseline (visit 1), week 4 (visit 2), week 16 (visit 3), week 28 (visit 4), and week 40 (visit 5) including p values from pairwise comparisons. (The figure is graphed using Stata version 16.0 (StataCorp LLC, College Station, TX, USA, <https://www.stata.com/>))


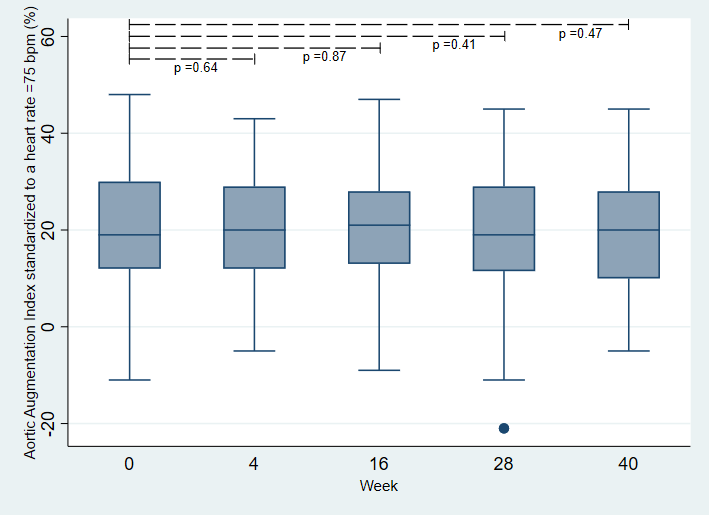


**Supplementary Figure 2 B:** Box plot of aortic augmentation index standardized to a heart rate of 75 bpm (AIx75) at baseline (visit 1), week 4 (visit 2), week 16 (visit 3), week 28 (visit 4), and week 40 (visit 5) including p values from pairwise comparisons. (The figure is graphed using Stata version 16.0 (StataCorp LLC, College Station, TX, USA, <https://www.stata.com/>))


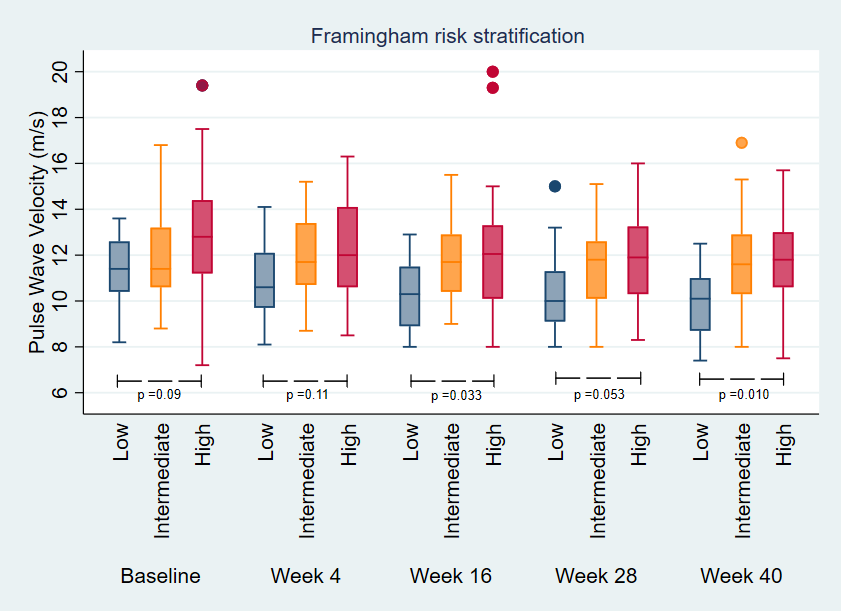


**Supplementary Figure 3:** Box plot of aortic PWV at baseline (visit 1), week 4 (visit 2), week 16 (visit 3), week 28 (visit 4), and week 40 (visit 5) according to Framingham risk stratification. (The figure is graphed using Stata version 16.0 (StataCorp LLC, College Station, TX, USA, <https://www.stata.com/>))


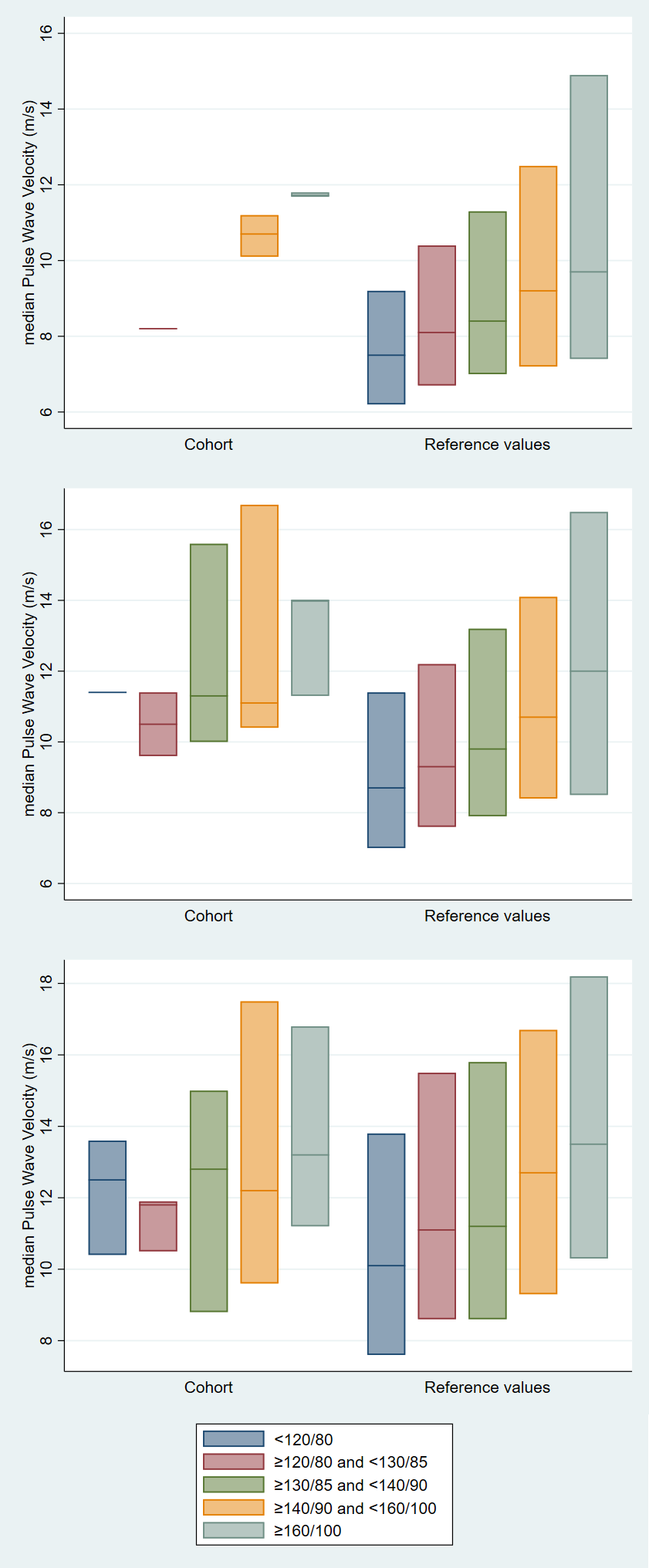
**Supplementary Figure 4:** Comparison of the median aortic PWV analysis at baseline according to age group (upper: 50–59 y, middle: 60–69 y, lower: ≥70 y) in the present cohort with the age- and blood pressure-matched individuals from the European reference population. (The figure is graphed using Stata version 16.0 (StataCorp LLC, College Station, TX, USA, <https://www.stata.com/>))


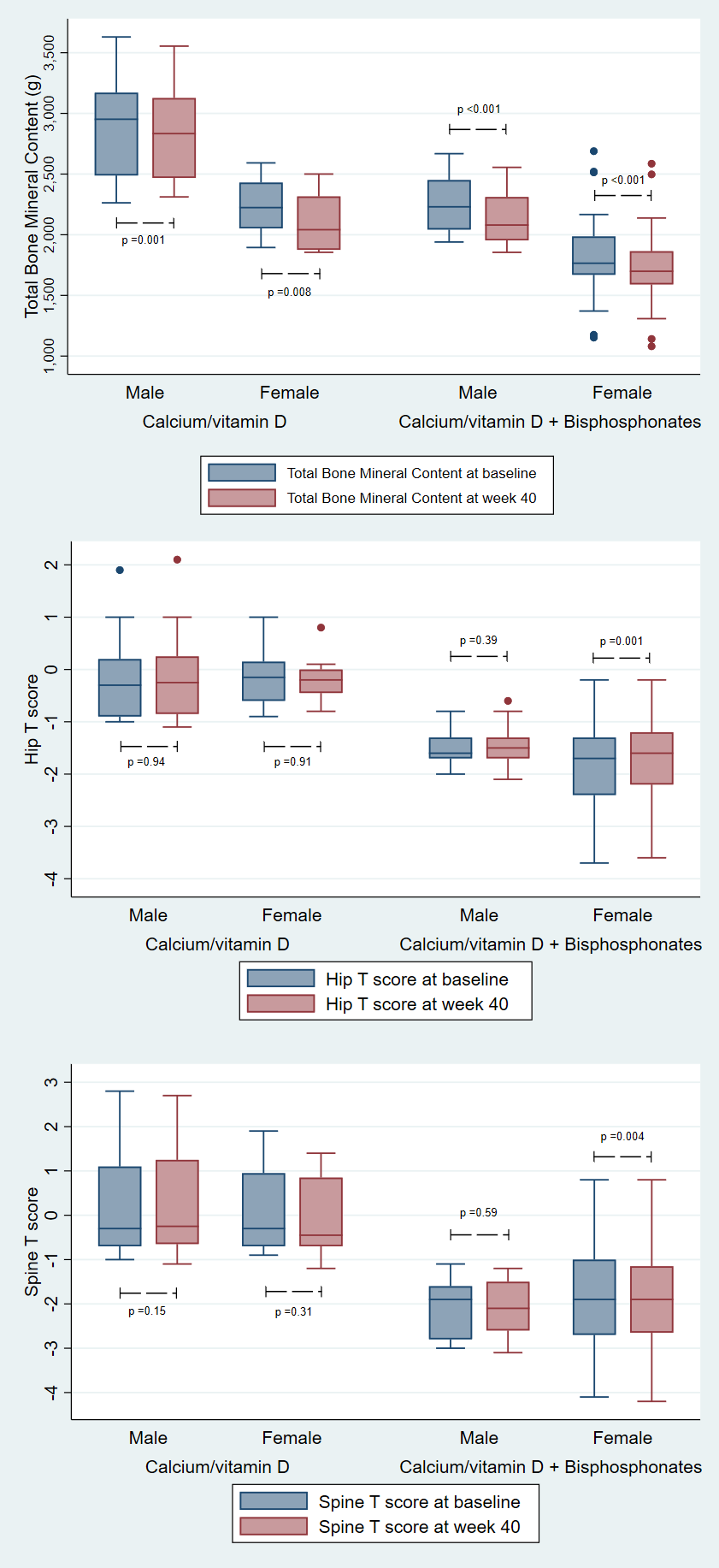
**Supplementary Figure 5:** Comparison of changes in total BMC as well as hip and spine T scores in those who were treated with calcium/vitamin D supplementation alone and those who were treated with calcium/vitamin D supplementation together with bisphosphonates. (The figure is graphed using Stata version 16.0 (StataCorp LLC, College Station, TX, USA, <https://www.stata.com/)>)
