# Supplementary material for: Prognostic impacts of glucocorticoid treatment in patients with polymyalgia rheumatica and giant cell arteritis
Source: Sci Rep. 2021 Mar 18;11:6220. doi: 10.1038/s41598-021-85857-4 (PMC7973518; doi:10.1038/s41598-021-85857-4)
Supplement: Supplementary file 2 — Supplementary Tables. [file 41598_2021_85857_MOESM2_ESM.docx]

**Prognostic impacts of Glucocorticoid treatment in patients with Polymyalgia Rheumatica and Giant Cell Arteritis.**

Amir Emamifar* (0000-0001-5329-1364), MD, PhD, [amir.emamifar@rsyd.dk](mailto:amir.emamifar@rsyd.dk), Torkell Ellingsen, (0000-0003-0426-4962), Clinical professor, PhD, [torkell.ellingsen@rsyd.dk](mailto:torkell.ellingsen@rsyd.dk), Anne Pernille Hermann, (0000-0001-6701-0981), Clinical associate professor, PhD, [pernille.hermann@rsyd.dk](mailto:pernille.hermann@rsyd.dk), Søren Hess (0000-0003-1249-133X), Clinical associate professor, [soren.hess@rsyd.dk](mailto:soren.hess@rsyd.dk), Oke Gerke (0000-0001-6335-3303), Professor, PhD, [Oke.Gerke@rsyd.dk](mailto:Oke.Gerke@rsyd.dk), Ziba Ahangarani Farahani, Senior consultant, [ziba.farahani2@rsyd.dk](mailto:ziba.farahani2@rsyd.dk), Per Syrak Hansen, Senior consultant, [Per.Syrak.Hansen@rsyd.dk](mailto:Per.Syrak.Hansen@rsyd.dk), Inger Marie Jensen Hansen (0000-0001-7283-9786), Former clinical associate professor, PhD, [imjh@carlhansen.dk](mailto:imjh@carlhansen.dk), Peter Thye-Rønn (0000-0002-7486-8086), Clinical associate professor, PhD, [Peter.Thye-Ronn@rsyd.dk](mailto:Peter.Thye-Ronn@rsyd.dk)

| **Supplementary Table 1: Disposition table** | | |
| --- | --- | --- |
|  | **N=77** | **Reason** |
| The number of patients who completed the study, n (%) | 69 (89.6%) | - |
| The number of patients withdrawn before visit 2 (week 4), n (%) | 4 (5.2%) | 3 not interested ,1 prednisolone side effect |
| The number of patients withdrawn before visit 3 (week 16), n (%) | 2 (2.6%) | 1 not interested, 1 died due to abdominal aorta aneurism |
| The number of patients withdrawn before visit 4 (week 28), n (%) | 2 (2.6%) | 1 not interested, 1 lost to follow-up |
| The number of patients withdrawn before visit 5 (week 40), n (%) | 0 | - |

| **Supplementary Table 2: Cardiovascular risk factors in the included patients.** | |
| --- | --- |
| Daily smoker, n (%) | 9 (11.7) |
| Systolic blood pressure, (mmHg) | 137 (127.5‐152) |
| Total cholesterol, mmol/L | 4.2±1.0 |
| HDL cholesterol, mmol/L | 1.4 (1.1-1.6) |
| Diabetes, n (%) | 6 (7.8) |
| Antihypertensive medicine, n (%) | 44 (57.1) |
| Lipid lowering medicine, n (%) | 27 (35.1) |
| Known vascular disease i.e myocardial infarct, peripheral vascular disease, stroke, n (%) | 12 (15.9) |

| **Supplementary Table 3: Correlation between PWV parameters at baseline.** | | | |
| --- | --- | --- | --- |
| **Variables** | **AIx** | **AIx75** | **PWV** |
| Aortic SP | 0.32^1^  **0.004** | 0.16^1^  0.17 | 0.40^2^  **<0.001** |
| Aortic DP | −0.14^1^  0.21 | 0.03^1^  0.80 | 0.21^2^  0.07 |
| Aortic PP | 0.47^1^  **<0.001** | 0.14^1^  0.22 | 0.26^2^  **0.027** |
| MAP | 0.05^1^  0.68 | 0.19^1^  0.10 | 0.33^2^  **0.004** |
| Aortic AP | 0.92^2^  **<0.001** | 0.69^2^  **<0.001** | 0.21^2^  0.07 |
| Aix | --- | 0.85^1^  **<0.001** | 0.15^2^  0.21 |
| AIx75 | 0.85^1^  **<0.001** | --- | 0.12^2^  0.30 |
| Pulse transit time | −0.11^2^  0.35 | −0.08^2^  0.48 | −0.89^2^  **<0.001** |
| HR | −0.24^1^  **0.036** | 0.19^1^  0.10 | −0.05^2^  0.66 |
| PWV | 0.15^2^  0.21 | 0.12^2^  0.30 | --- |
| SP: Systolic pressure, DP: Diastolic pressure, PP: Pulse pressure, MAP: Mean arterial pressure, AP: Augmented pressure, AIx: Aortic augmentation index, AIx75: Aortic augmentation index standardized to a heart rate of 75 beats per minute, HR: Heart rate, PWV: Pulse wave velocity  1. Pearson's correlation  2. Spearman's rank correlation | | | |

| **Supplementary Table 4: Comparison of baseline PWV, AIx, AIx75 by TAB, clinical diagnosis and 18F-FDG PET/CT results.** | | | | | | |
| --- | --- | --- | --- | --- | --- | --- |
| **Variables** | **PWV** | **P-value** | **AIx, %** | **P-value** | **AIx75, %** | **P-value** |
| TAB  Negative  Positive | 11.6 (10.6-13.2)  12.4 (11.4-13.9) | 0.34^1^ | 21.3±11.9  24.1±10.2 | 0.55^3^ | 21.3±12.1  21.3±14.8 | 0.99^3^ |
| Clinical diagnosis  Pure PMR  Pure GCA  Concomitant PMR and GCA | 11.8 (10.6-13.6)  13.9 (11.4-14.4)  11.8 (11.4-13) | 0.56^2^ | 20.8 ±11.9  21±12.5  27.2±13.8 | 0.30^4^ | 20.6±11.6  20±15.5  26.9±16.4 | 0.32^4^ |
| 18F-FDG PET/CT cut off ≥3  Neither PMR nor GCA activity  PMR activity  GCA activity  PMR and GCA activity | 11.5 (10.7-13.3)  12 (10.7-13.8)  13.2 (12.1-14.4)  9.6 (9.6-9.6) | 0.27^2^ | 22.8±15.2  21.0±11.1  17.5±6.4  43 ±0 | 0.31^4^ | 22.4±14.3  20.9±11.4  12±9.9  48±0 | 0.11^4^ |
| 18F-FDG PET/CT cut off ≥2  Neither PMR nor GCA activity  PMR activity  GCA activity  PMR and GCA activity | 11.4 (10.5-11.6)  11.9 (10.7-13.6)  11.8 (11.4-14.4)  13.2 (10.1-13.9) | 0.56^2^ | 21.9±15.9  20.4±11.5  33.3±13.3  25.4±12.7 | 0.24^4^ | 20.9±14.5  20.3±11.4  33.7±12.9  24.7±16.2 | 0.26^4^ |
| VGS: Visual Grading Scale  1. Wilcoxon rank-sum test  2. Kruskal–Wallis test  3. Student’s t-test  4. Analysis of variance | | | | | | |

| **Supplementary Table 5: Correlation analysis between PWV, AIx, AIx75, and clinical data.** | | | |
| --- | --- | --- | --- |
| **Variables** | **PWV** | **AIx, %** | **AIx75, %** |
| Age | 0.25  **0.030^1^** | 0.25  **0.027^2^** | 0.11  0.32^2^ |
| Charlson comorbidity index score | 0.21  0.07^1^ | 0.17  0.15^1^ | 0.02  0.84^1^ |
| Patients pain VAS score | 0.05  0.71^1^ | −0.03  0.82^1^ | −0.02  0.87^1^ |
| Patients global VAS score | −0.02  0.86^1^ | −0.05  0.67^1^ | 0.09  0.47^1^ |
| Physician global VAS score | −0.02  0.84^1^ | −0.19  0.13^1^ | −0.10  0.41^1^ |
| Left systolic blood pressure | 0.25  **0.032^1^** | −0.05  0.67^1^ | −0.10  0.37^1^ |
| Left diastolic blood pressure | 0.19  0.10^1^ | −0.21  0.06^1^ | −0.05  0.65^1^ |
| Right systolic blood pressure | 0.28  **0.018^1^** | 0.02  0.89^1^ | −0.08  0.50^1^ |
| Right diastolic blood pressure | 0.25  **0.033^1^** | −0.22  0.06^1^ | −0.08  0.49^1^ |
| Left radial pulse, per minute | −0.05  0.65^1^ | −0.24  **0.031^1^** | 0.21  0.07^1^ |
| Right radial pulse, per minute | −0.05  0.68^1^ | −0.26  0.020 | 0.19  0.10^1^ |
| ESR | −0.01  0.92^1^ | 0.23  **0.046^1^** | 0.31  **0.007^1^** |
| CRP | −0.12  0.32^1^ | −0.04  0.75^1^ | 0.12  0.29^1^ |
| Fibrinogen | 0.05  0.67^1^ | 0.04  0.71^1^ | 0.09  0.45^1^ |
| HDL | −0.16  0.23^1^ | −0.18  0.15^2^ | −0.20  0.13^2^ |
| LDL | −0.18  0.17^1^ | −0.16  0.21^1^ | −0.30  **0.017^1^** |
| PMR score | 0.10  0.37^1^ | 0.04  0.70^1^ | 0.08  0.47^1^ |
| GCA score | 0.11  0.36^1^ | 0.13  0.27^1^ | 0.08  0.49^1^ |
| Cumulative prednisolone dose | 0.10  0.44^1^ | 0.06  0.62^1^ | 0.21  0.07^1^ |
| Total number of relapse | −0.22  0.07^1^ | 0.09  0.46^1^ | 0.16  0.18^1^ |
| 1. Spearman's rank correlation  2. Pearson's correlation | | | |

| **Supplementary Table 6: The median (10^th^-90^th^) of aortic PWV at baseline in the present cohort according to age groups and blood pressure.** | | | | | | | | | | | | | | |
| --- | --- | --- | --- | --- | --- | --- | --- | --- | --- | --- | --- | --- | --- | --- |
|  | **Blood Pressure category** | | | | | | | | | |  | |  | |
|  | <120/80 | | ≥120/80 and <130/85 | | ≥130/85 and <140/90 | | ≥140/90 and <160/100 | | ≥160/100 | | DM | | CVD | |
| **Age category** | N* | Median (10^th^-90^th^) | N | Median (10^th^-90^th^) | N | Median (10^th^-90^th^) | N | Median (10^th^-90^th^) | N | Median (10^th^-90^th^) | N | Median (10^th^-90^th^) | N | Median  (10^th^-90^th^) |
| 50-59 | 0 | - | 1 | 8.2  (8.2-8.2) | 0 | - | 3 | 10.7 (10.1-11.2) | 2 | 11.7  (11.7-11.8) | 0 | - | 0 | - |
| 60-69 | 1 | 11.4  (11.4-11.4) | 3 | 10.5 (9.6-11.4 | 8 | 11.3 (10-15.6) | 7 | 11.1  (10.4-16.7) | 3 | 14  (11.3-14) | 1 | 11.6  (11.6 -11.6) | 2 | 13.1  (12.6-13.6) |
| ≥70 | 6 | 12.5  (10.4-13.6) | 6 | 11.8 (10.5-11.9) | 10 | 12.8  (8.8-15) | 20 | 12.2  (9.6-17.5) | 7 | 13.2  (11.2-16.8) | 5 | 11.5  (10.6-19.4) | 10 | 12.4  (7.2-14.4) |
| DM: Diabetes Mellitus, CVD: Overt Cardiovascular Disease  *numbers of observation | | | | | | | | | | | | | | |
